# Individuals with high bone mass have an increased prevalence of radiographic knee osteoarthritis

**DOI:** 10.1016/j.bone.2014.10.015

**Published:** 2015-02

**Authors:** S.A. Hardcastle, P. Dieppe, C.L. Gregson, N.K. Arden, T.D. Spector, D.J. Hart, M.H. Edwards, E.M. Dennison, C. Cooper, A. Sayers, M. Williams, G. Davey Smith, J.H. Tobias

**Affiliations:** a Musculoskeletal Research Unit, School of Clinical Sciences, University of Bristol, UK; b MRC Integrative Epidemiology Unit, School of Social and Community Medicine, University of Bristol, UK; c University of Exeter Medical School, Exeter, UK; d Oxford NIHR Musculoskeletal Biomedical Research Unit, University of Oxford, Oxford, UK; e MRC Lifecourse Epidemiology Unit, University of Southampton, Southampton, UK; f Arthritis Research UK (ARUK) Centre for Sports, Exercise and Osteoarthritis, University of Oxford, Nuffield Orthopaedic Centre, Oxford, UK; g Department of Twin Research and Genetic Epidemiology, King's College London, London, UK; h NIHR Nutrition Biomedical Research Centre, University of Southampton, Southampton, UK; i Department of Radiology, North Bristol NHS Trust, Bristol, UK

**Keywords:** Osteoarthritis, DXA, Bone mineral density, High bone mass

## Abstract

We previously reported an association between high bone mass (HBM) and a bone-forming phenotype of radiographic hip osteoarthritis (OA). As knee and hip OA have distinct risk factors, in this study we aimed to determine (i) whether HBM is also associated with knee OA, and (ii) whether the HBM knee OA phenotype demonstrates a similar pattern of radiographic features to that observed at the hip.

HBM cases (defined by DXA BMD Z-scores) from the UK-based HBM study were compared with unaffected family controls and general population controls from the Chingford and Hertfordshire cohort studies. A single blinded observer graded AP weight-bearing knee radiographs for features of OA (Kellgren–Lawrence score, osteophytes, joint space narrowing (JSN), sclerosis) using an atlas. Analyses used logistic regression, adjusting *a priori* for age and gender, and additionally for BMI as a potential mediator of the HBM–OA association, using Stata v12.

609 HBM knees in 311 cases (mean age 60.8 years, 74% female) and 1937 control knees in 991 controls (63.4 years, 81% female) were analysed. The prevalence of radiographic knee OA, defined as Kellgren–Lawrence grade ≥ 2, was increased in cases (31.5% *vs.* 20.9%), with age and gender adjusted OR [95% CI] 2.38 [1.81, 3.14], p < 0.001. The association between HBM and osteophytosis was stronger than that for JSN, both before and after adjustment for BMI which attenuated the ORs for knee OA and osteophytes in cases *vs.* controls by approximately 50%.

Our findings support a positive association between HBM and knee OA. This association was strongest for osteophytes, suggesting HBM confers a general predisposition to a subtype of OA characterised by increased bone formation.

## Highlights

• We examined associations between high bone mass and radiographic knee osteoarthritis (OA).
• High bone mass cases had an increased prevalence of knee OA compared with controls.
• The OA phenotype in high bone mass is characterised by osteophytosis.
• Body mass index is a partial mediator of the high bone mass–OA association.

## Introduction

The nature of the relationship between bone mineral density (BMD) and osteoarthritis (OA) remains a topic of debate [1]. While epidemiological studies have consistently demonstrated an association between higher BMD and both prevalent [2–5] and incident [6–8] radiographic OA of the large joints, the mechanisms behind these associations remain unclear; understanding these mechanisms will be key to translating research findings into therapeutic benefit [1]. To address this question from a novel perspective, we set out to investigate the prevalence and phenotype of OA in our cohort of high bone mass (HBM) individuals [9], compared with a control group. HBM individuals have extreme elevations in BMD likely to be genetically determined [9,10] and thus present from early adulthood, constituting a unique population for the investigation of causal pathways between BMD and OA. We have recently shown that HBM is associated with both an increased prevalence of self-reported joint replacement [11], and an increased prevalence of radiographic hip OA with a predominance of bone-forming features (osteophytosis and subchondral sclerosis) [12]. HBM is also associated with other characteristics which may potentially contribute to a higher risk of OA, including increased body mass index (BMI) [13].

While hip and knee OA both increase with age [14], evidence suggests that OA at these two joint sites has different determinants [15]. In particular, whereas local mechanical factors acting at the joint level may be more important for hip OA [16], knee OA has a stronger association with OA at other joint sites such as the hand [14,17] suggesting a more generalised systemic predisposition to the disease. The concept of knee and hip OA as different diseases is supported by the fact that hip OA appears to be more heritable than knee OA [18], and genetic studies indicate little genetic correlation between the two disorders [19]. The role of specific risk factors for OA at these two joint sites is also thought to differ; for example, the relationship between obesity and OA is reported to be stronger at the knee compared with the hip [15,20,21], and knee OA is more prevalent in females than males [14]. We therefore wished to establish whether any relationship between HBM and OA of the knee is similar to that previously observed at the hip.

The aim of this study was to investigate radiographic knee OA in our HBM population, determining i) whether HBM is associated with an increased prevalence of radiographic knee OA, ii) the phenotype of knee OA in HBM compared with controls in terms of individual radiographic features, and iii) the role of potential mediators such as BMI. We hypothesized that, in line with our previous findings and evidence from general population studies, HBM would be associated with a bone-forming phenotype of radiographic knee OA.

## Methods

### The HBM population

HBM cases were recruited as part of the UK-based HBM study, a multi-centre observational study of adults with unexplained HBM. Index cases were initially identified by screening DXA databases for T and/or Z-scores ≥ + 4. All DXA images were inspected by trained clinicians in order to exclude scans with artefactual elevation of DXA BMD, resulting in 49.4% of scans being excluded due to degenerative disease/osteoarthritis/scoliosis, and a further 15.5% for other reasons including surgical/malignant/Pagetic artefacts etc. Then, in order to identify generalised HBM, the HBM index case definition was refined to either a) L1 Z-score ≥ + 3.2 plus total hip Z-score ≥ + 1.2 or b) total hip Z-score ≥ + 3.2 plus L1 Z-score ≥ + 1.2. A + 3.2 threshold was consistent with the only published precedent for identifying HBM using DXA [22]. L1 Z-score was used to avoid misclassifying individuals with lower lumbar OA as having HBM [9,23]. Z rather than T-score limited age bias.

Further HBM cases were identified through DXA assessment of the relatives and spouses of index cases. In first-degree relatives, HBM was defined as a summed L1 Z-score plus total hip Z-score ≥ + 3.2. 41% of relatives screened were affected and combined with HBM index cases, with remaining unaffected first-degree relatives/spouses forming a family control group. Full details of this DXA database screening and recruitment have been previously reported [9]. Assessments, including a structured interview and clinical examination, were identical in both HBM cases and controls, and AP weight-bearing knee X-rays were performed in all participants according to local protocols at each centre. Recruitment ran from July 2005–April 2010. Written informed consent was obtained from all participants in line with the Declaration of Helsinki [24] and the study was approved by the Bath multi-centre Research Ethics Committee (REC) and each NHS local REC. For this study, HBM cases were then categorised into 5-year age bands by gender, prior to selection of additional population controls, using age and gender-stratified random sampling.

### Population-based controls

Population controls were selected from the Chingford 1000-women study (ChS) and Hertfordshire cohort study (HCS). The ChS is a prospective longitudinal female population-based cohort which initially recruited 1003 women aged 45–64 from the age/sex register of a general practice in Chingford, North-East London [2]; 20-year follow-up has recently taken place. AP knee radiographs were obtained in years 1, 5, 10, 15 and 20. Controls, according to age at the time of X-ray, were randomly sampled in a 2:1 ratio with HBM female cases for each age band apart from the lower (40–50 years) and upper (> 80) bands (3:1). A single radiograph per participant was included in our study, with controls in the upper age bands selected first to ensure sufficient numbers of available films.

The HCS [25] recruited approximately 3000 men and women born in Hertfordshire between 1931 and 1939 and still resident there in 1998–2003. Recently a subset of HCS participants were recruited into the European Project on Osteoarthritis (EPOSA) [26]; these individuals (207 men and 203 women now aged between 71.5 years and 80.6 years) had AP pelvis +/− weight-bearing knee X-rays performed during 2011. These individuals were randomly sampled 2:1 with HBM cases within each appropriate age band (70–75, 75–80 and > 80).

### Assessment of radiographs

All available case and control radiographs were pooled for assessment. Files were automatically relabelled with anonymised codes, and presented in a random order to ensure blinding of the assessor. Radiographs were graded by a single observer (SH) following focussed radiological training. X-ray images were viewed and quantitative measurements made using open source ImageJ software [27]; semi-quantitative assessments were recorded within a Microsoft Access database.

Each knee was first assigned a global Kellgren–Lawrence OA grade [28], followed by semi-quantitative grading of individual radiographic features of OA using an established atlas [29] (Table 1); the presence or absence of chondrocalcinosis (previously shown to be associated with radiographic knee OA and osteophytosis [30]) was also noted (0–1). Each of these features was recorded separately in the medial and lateral compartments. For knees with OA (KL grade ≥ 2) only, the compartments affected (medial/lateral/both) were recorded. As all radiographs were performed AP, only the tibiofemoral joint was assessed.

A Kellgren–Lawrence grade of 2 (at least 1 definite osteophyte) defined the presence of OA in the main analysis; however, because definitions of knee OA vary between studies [31], results are also shown using a KL grade threshold of ≥ 3 (osteophytes and joint space narrowing). Categorical scores for the individual radiographic features were converted to binary variables for analysis (Table 1). Quantitative measurement of minimum medial compartment joint space width (JSW) was made within Image J, using the line tool, facilitated by a simple macro. JSW measurement was limited to the medial compartment only, as this measure is poorly reproducible in the lateral compartment of the knee [32–34]. As differences in radiographic protocols between studies can potentially result in varying degrees of magnification of the X-ray image, we could not reliably compare quantitative measures between studies; analysis of measured JSW was therefore limited to the HBM cases and family controls only.

Image quality was rated by the operator at the time of assessment (good, poor, very poor), with very poor X-rays, judged in terms of penetration and/or resolution, excluded. If the X-ray was grossly rotated or tilted, this was recorded. Joint replacements were recorded and these knees excluded from the main analysis (a sensitivity analysis was later performed including these X-rays). At the end of the study 126 randomly selected knees were re-graded by the primary observer to assess intra-rater repeatability. Intra-rater kappa values for the above listed binary variables were all ≥ 0.78 except subchondral sclerosis (0.39); however, subchondral sclerosis was rarely seen. The intra-rater kappa for knee compartment involvement (medial/lateral/both) was 0.84. The intra-class correlation coefficient (ICC) for minimum measured JSW was 0.98.

### Assessment of covariates

Values for age, gender and body mass index (BMI) were obtained from each pre-existing study dataset. Age was defined by the time of X-ray. BMI was calculated as weight (kg)/height (metres^2^) using the closest available weight and height measurements to the time of the X-ray. Body composition data, derived from total body DXA scans, were available in a proportion of HBM cases and family controls using methods previously described [13]. As total body DXA scans in the HBM group were performed on both GE Lunar Prodigy and Hologic Discovery DXA scanners depending on recruitment centre, validated cross-calibration equations were applied for all bone and soft tissue regions of interest [35]. Additional height, weight and BMI measures obtained at the time of total body DXA were also available in this group.

### Statistical analysis

Demographic statistics for the HBM cases and each control population were summarised as mean (SD) for continuous variables and counts (percentages) for categorical variables. In this case–control analysis, categorical variables were initially cross-tabulated and percentages calculated: the chi-squared (χ^2^) test was used to assess the association between binary variables, and the unpaired t-test to compare mean values for continuous JSW. Associations between HBM case status and binary radiographic OA outcomes were then analysed using generalised estimating equations (GEE) with a logistic link function (logistic regression allowing for clustering of observations within individuals, i.e. right/left knees). Analyses were adjusted for the *a priori* confounders age and gender, and then additionally for BMI as a potential mediator. Odds ratios before and after adjustment are presented with 95% confidence intervals (95% CI), and p values from Wald significance tests. GEE using an identity link function (linear regression allowing for clustering) was used to compare medial compartment minimum JSW (mm) in HBM cases and family controls, adjusting for confounders. The possible mediating role of BMI was then more formally explored using a binary mediation approach with a probit model, and additionally by adjusting for the different components of body mass (fat mass, lean mass etc.) in turn. Analyses were repeated stratified by gender.

Pre-planned sensitivity analyses comprised: i) exclusion of poor quality/rotated/tilted X-rays, ii) a “person-level” analysis of the worst knee in each individual, iii) adding radiographic knee replacements to the dataset, assuming these were performed for OA, iv) excluding HBM cases/controls with self-reported inflammatory arthritis, and v) restricting the analysis to those HBM cases meeting the index case definition at the hip. Data were analysed using Stata release 12 statistical software (StataCorp, College Station, TX, USA).

## Results

### Participant selection and characteristics

**Fig. 1:**
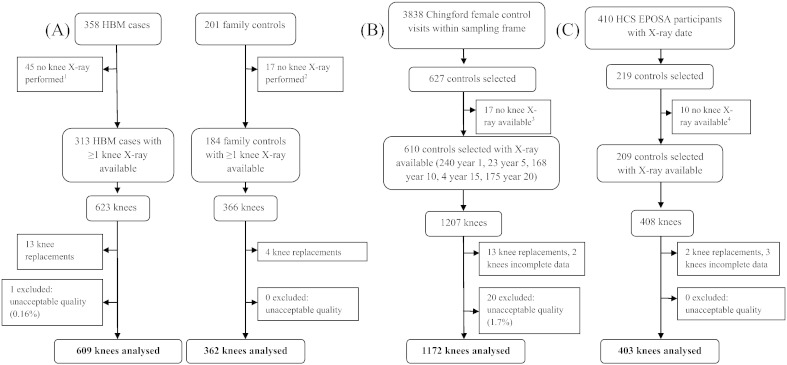
Flow diagram summarising selection of radiographs for inclusion in the study. (A) Selection of high bone mass (HBM) case and family control X-rays (process of recruitment to study described previously). (B) Selection of Chingford study female control X-rays. (C) Selection of HCS EPOSA male and female control X-rays. ^1^Reason recorded for missing X-rays in HBM cases: unable to travel (n = 7), no X-rays at study centre (n = 10), unable to attend/wait/comply (n = 3), patient declined (n = 6), reside abroad (n = 2), bilateral knee replacements (n = 6), not done (reason unknown) (n = 10). ^2^Reason recorded for missing X-ray in family controls: unable to travel (n = 1), did not continue in study (n = 1), no X-rays at study centre (n = 4), unable to attend/wait/comply (n = 3), patient declined (n = 2), bilateral knee replacements (n = 3), reason unknown (n = 3). ^3^Reason recorded for missing X-ray in Chingford controls: did not continue in study (n = 3), file corrupted (n = 2), unknown (n = 12). ^4^Reason recorded for missing X-ray in HCS EPOSA controls: bilateral knee replacements (n = 6), unknown (n = 4).

Fig. 1 summarises the selection of radiographs for inclusion in our study. 21 knee joints (n = 1 case, 20 controls) were excluded from the outset due to unacceptable image quality. Knee replacements were also excluded (n = 13 cases, 19 controls). 2546 knees from 1302 individuals were included in the primary combined analysis comprising 609 HBM case knees, 362 family control knees, 1172 ChS control knees and 403 HCS control knees. 1244 individuals contributed two knees to the analysis and 58 individuals contributed only one knee. Table 2 summarises the demographics of the study population. HBM cases were slightly younger than the combined controls (mean 60.8 years *vs.* 63.4 years), with a lower proportion of females (74.3% *vs.* 81.3%). As expected, HBM cases had substantially higher values for standardised BMD at both the hip and lumbar spine compared with controls. Mean BMI was also greater in cases than controls (30.6 *vs.* 27.3 kg/m^2^).

### HBM case status and knee OA: unadjusted analyses

The prevalence of the different OA outcomes is shown for HBM cases, each separate control group, and all control groups combined (Table 3). The prevalence of radiographic knee OA (defined as KL grade ≥ 2) was 31.5% in HBM cases and 20.9% in the combined controls (p < 0.001); as expected this was identical to the prevalence of any osteophyte (≥ grade 1). Moderate osteophytes (≥ grade 2), moderate JSN (≥ grade 2) and chondrocalcinosis were also more prevalent in HBM cases. No difference was observed between the HBM cases and the combined control group in the prevalence of milder JSN (≥ grade 1) or subchondral sclerosis (which was only seen infrequently). Knee OA defined as KL grade ≥ 3 was also more prevalent in HBM cases.

### HBM case status and knee OA: analyses adjusted for age and gender

Following age and gender adjustment, radiographic knee OA remained strongly associated with HBM, with an odds ratio [95% CI] of 2.38 [1.81,3.14], p < 0.001 (model 2, Table 4). Of the individual radiographic OA features, the largest odds ratios were seen for the osteophyte variables (e.g. OR 2.40 [1.69,3.41] for moderate osteophyte, p < 0.001). The odds of any JSN did not differ between cases and controls (1.18 [0.86,1.62], p = 0.299); however, moderate JSN remained more frequent in the HBM group (1.95 [1.20,3.18], p = 0.007). The odds of chondrocalcinosis (1.65 [1.02,2.66], p = 0.042) was also greater in the HBM group, but did not explain the association between HBM and knee OA (OR 2.33 [1.77,3.09] for knee OA (KL ≥ 2) in HBM cases *vs.* controls after adjustment for the presence of chondrocalcinosis). More severe knee OA (KL ≥ 3) was also associated with HBM case status (1.98 [1.39,2.82], p < 0.001), albeit with a slightly smaller odds ratio than that seen with our primary definition.

These analyses were repeated comparing HBM cases with each of the separate control groups, and then stratified by gender. Adjusted findings were broadly similar when analyses were restricted to HBM cases *vs.* family controls (Supplementary Table 1). Minimum measured JSW in the medial compartment did not differ between the HBM cases and family controls (mean difference 0.02 mm [− 0.15,0.20], p = 0.817, adjusted for age and gender). Comparing HBM female cases with ChS controls alone (Supplementary Table 2), and older HBM cases with HCS controls (Supplementary Table 3) also gave broadly similar results. When restricted to females only (Supplementary Table 4), estimates for most variables were essentially unchanged with respect to the main analysis. In males (Supplementary Table 5), odds ratios for several outcomes in HBM cases increased, including knee OA, osteophytes, JSN and subchondral sclerosis. However, confidence intervals were widened, reflecting the smaller numbers of males included in our study, and no formal evidence of a gender interaction was seen (interaction p value 0.53 for KL ≥ 2, with age adjustment).

### Effect of adjusting for BMI

**Fig. 2:**
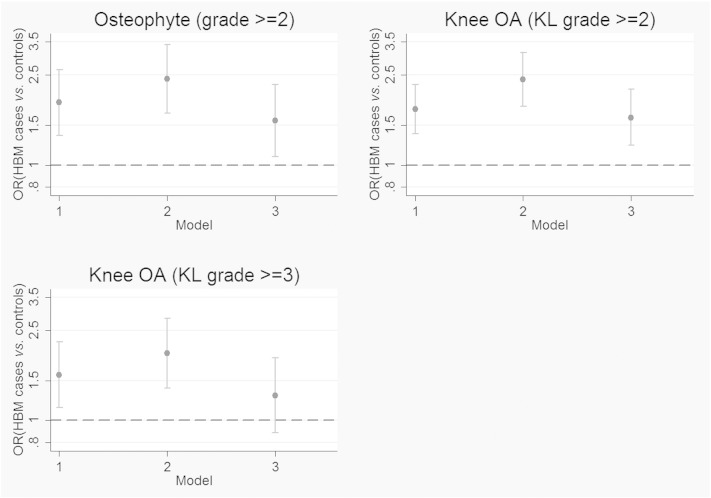
Effect of BMI adjustment on association between HBM case status and osteophyte/knee OA. OR = odds ratio in HBM cases *vs.* combined controls; error bars show 95% confidence interval. Model 1 = unadjusted, model 2 = adjusted for age and gender, model 3 = adjusted for age, gender and BMI. N (total no. knee joints analysed) = 2546 (609 HBM cases, 1937 controls). Dashed line shows OR of 1 (ie. no difference between the groups).

**Fig. 3:**
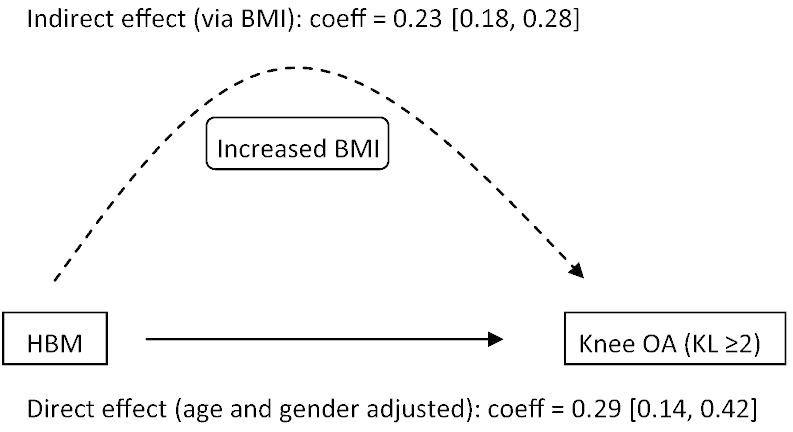
Mediation analysis examining direct and indirect association of HBM with radiographic knee OA (probit model). Knee OA defined as KL grade ≥ 2. N = 609 HBM cases, 1937 controls. Coefficient for total effect of HBM on knee OA (probit model) = 0.52 [0.37, 0.65]. Indirect effect (dashed arrow) represents the proportion of the effect estimated to be mediated by BMI. Ratio of indirect:direct effect estimated as 0.81, with proportion of total effect mediated by BMI 0.45 (45%). Estimates and 95% confidence intervals [square brackets] were obtained using a non-parametric bootstrap procedure with resampling for 1000 iterations. Confidence intervals are based on 2.5th and 97.5th percentiles of the bootstrapped distribution.

Further adjustment for BMI resulted in partial attenuation of the age and gender adjusted odds ratios for moderate osteophytes and knee OA in HBM cases *vs.* controls (Fig. 2). The association between HBM case status and knee OA defined as KL ≥ 3 was fully attenuated (Supplementary Table 6). These results suggest that BMI is a partial mediator of the HBM–OA association at the knee. Mediation analysis was used to explore this possibility further. By comparing the coefficients for the direct and indirect (via BMI) pathways, it was estimated that 45% of the association between HBM case status and knee OA is mediated by BMI (Fig. 3).

Total body DXA data were available in 190 HBM cases (mean age 61 years, 75.8% female) and 121 family controls (mean age 55 years, 46.3% female). We used these data to explore the effect of adjusting for different body mass compartments on the HBM–OA relationship (Supplementary Table 7). Using our age and gender adjusted model, adjustment for height and then either weight, or fat and lean mass, produced similar degrees of attenuation compared with BMI adjustment. When each parameter was added individually to the regression model, fat mass resulted in the greatest attenuation of the HBM–OA association (similar to that for BMI) whereas lean mass, despite representing a greater proportion of overall mass, appeared less important. If anything, adjustment for individual fat compartments (trunk, peripheral [arms and legs], android and gynoid) led to less attenuation than adjustment for total fat mass, suggesting that overall weight and fat mass are more important than fat distribution.

### Pattern of knee OA in HBM cases *vs.* controls

Patterns of knee compartment involvement were examined first in all knees with KL grade ≥ 2, and then in knees with KL ≥ 3 (definite osteophyte plus narrowing) only (Table 4), excluding those HBM cases with a self-reported history of inflammatory arthritis. Predominant medial compartment disease was the most prevalent pattern in both HBM cases and controls, in whom OA patterns were similar. If anything, amongst narrowed knees, the proportion of medial compartment disease was slightly greater in HBM cases compared with the control group (p = 0.037); however, this association did not persist after age and gender adjustment.

### Sensitivity analyses

315 X-rays (15 HBM case knees, 300 control knees) were considered to be poor quality in terms of resolution/penetration/artefact etc. A further 210 knees (58 case knees, 152 control knees) had significant rotation or tilt. Excluding all of these knees from the analysis did not materially affect the HBM–knee OA association observed (OR 2.45 [1.82,3.30], p < 0.001 for KL ≥ 2, adjusted for age and gender). Findings for JSN (most likely to be affected by tilt) were also essentially unchanged (data not shown). A person-level analysis, in which the worst knee per participant was analysed, also gave similar results (Supplementary Table 8). Radiographic knee replacements were excluded from the main analysis; including these knees (n = 32) and grading them as KL = 4 resulted in marginally increased odds ratios for knee OA in HBM (Supplementary Table 9). A small number of HBM cases and family controls reported a history of inflammatory arthritis: excluding these knees from the overall combined analysis (n = 35 HBM case knees, 4 family control knees) again did not materially change our findings (OR 2.33 [1.76,3.09], p < 0.001 for KL ≥ 2, adjusted for age and gender). Data on inflammatory arthritis were not available for the population controls. Restricting the analysis to those HBM cases meeting our index definition on the basis of their hip BMD alone (total hip Z-score ≥ + 3.2, n = 268 knees) resulted in an increased age and gender-adjusted OR for knee OA (OR 3.19 [2.21, 4.62], p < 0.001 for KL ≥ 2 in HBM cases *vs.* controls).

## Discussion

Our data indicate an increased prevalence of radiographic knee OA in HBM individuals compared with controls, consistent with existing epidemiological evidence that increased BMD is a risk factor for OA at this joint [2,5,6,8,36]. As we hypothesized, associations with HBM were stronger for the osteophyte variables compared with both semi-quantitative and measured JSN, particularly in models adjusted for BMI, and the stronger association we observed between HBM and knee OA defined as KL ≥ 2 (osteophytosis) versus KL ≥ 3 (osteophytosis plus JSN) is likely to be a further reflection of this. However, we found little evidence of an association between HBM and subchondral sclerosis, possibly explained by the very low prevalence of this feature and/or the difficulty of assessing its presence or absence on simple visual inspection of radiographs. A positive association between HBM and chondrocalcinosis was also seen; however, while chondrocalcinosis was also associated with radiographic knee OA, it did not explain the HBM–OA association observed.

Adjusting for BMI attenuated the HBM–knee OA association by approximately 50%, suggesting that the HBM–OA association at the knee is partly mediated through increased BMI. We previously reported that HBM is associated with a metabolic phenotype comprising greater BMI [9], and increased fat mass in women [13]; similar body composition changes have been observed in association with OA [37,38]. The primary mechanism by which weight/BMI contributes to OA in load-bearing joints has not been fully established; in particular the relative contribution of increased joint loading due to greater body weight [14,15] versus the effects of circulating metabolic factors such as adipokines [39] remains to be determined. It is interesting that, in our total body DXA analyses, adjusting for fat mass led to greater attenuation of the HBM–OA association compared with lean mass adjustment. This is consistent with some previous studies suggesting that increased fat mass relative to lean mass may be particularly associated with OA at the knee [40,41], possibly indicating a role for metabolic factors over and above body weight in determining OA risk. There may be gender differences in these relationships, for example a recent study suggested that fat mass and lean mass may be more important determinants of knee OA in women and men respectively [42]; this observation may therefore reflect the greater proportion of women in our study. Unfortunately numbers of males with total body DXA data did not permit gender-stratified analysis. It should be noted that these analyses were restricted to a subgroup of HBM cases and family controls only, resulting in limited statistical power.

We previously reported, based on a study of this same population, that HBM is associated with a bone-forming (osteophyte-predominant) phenotype of hip OA [11]. Unlike in the present study, adjusting for BMI resulted in only minimal attenuation of the association between HBM and hip OA compared with age and gender adjustment alone, consistent with evidence that BMI is less strongly associated with hip than knee OA. However interestingly, following age, gender and BMI adjustment, overall odds ratios for OA in HBM cases *vs.* combined controls were similar at the hip (1.52 [1.09,2.11]) [12] and the knee (1.62 [1.22,2.16]), suggesting that the increased risk of OA conferred as a direct result of HBM (independent of BMI) is similar at both joint sites. These findings suggest firstly that increased BMD is an important risk factor for OA at both the hip and knee, and secondly that increased bone formation, as evidenced by osteophytosis, drives this association at both joint sites.

Extreme BMD elevation, as seen in our HBM cases, is likely to be primarily genetically determined. Therefore an important consideration is the extent to which HBM individuals may be predisposed to “standard” (previously termed “common garden-variety” [43]) OA, as opposed to a distinct OA subtype arising from the pleiotropic effects of rare genetic variants. The former would have greater implications for our understanding of OA in the general population. We explored this question by examining the compartmental distribution of knee OA in our study population; whereas knee OA is expected to predominantly affect the medial tibiofemoral joint (subject to greater loading [44]), many rarer inflammatory, erosive or genetic forms of OA have a predilection for the lateral compartment [43]. Our observation that predominantly medial compartment knee OA was by far the most common pattern in both the HBM and control groups supports the view that HBM is associated with an increased risk of “standard” OA, and that the mechanisms underlying this relationship are applicable to the wider population.

Plausible mechanisms that might contribute to a bone-forming phenotype in HBM include upregulation of the Wnt signalling pathway. Activating mutations of this pathway are known to result in HBM [22], and evidence is accumulating for a role of altered Wnt signalling in OA [45–47]. Wnt signalling is also known to play a key role in the anabolic response of bone to mechanical loading, as evidenced by animal studies [48,49], and blockade of the Wnt signalling pathway inhibitor DKK-1 has been shown to promote osteophytosis in mice [50]. While the precise genetic basis of HBM in the majority of cases remains to be determined [51], and is the subject of ongoing studies, it is interesting to note that a genome-wide association study in this population showed overrepresentation of SNPs associated with BMD in the wider population including loci in Wnt pathway genes [10].

Our study has a number of limitations. We lack temporal data, so the direction of causality cannot be formally assessed; nevertheless, we assume the onset of genetically determined HBM would predate the onset of OA (a disease of later life) in this population. However, it is theoretically possible that OA features within the DXA field (e.g. lumbar osteophytosis) could lead to artefactual elevation of measured BMD, with the potential to induce a spurious association between HBM and OA if spine and knee OA are correlated as part of a “generalised OA” phenotype. As discussed, every effort was made to avoid such misclassification of HBM status through both inspection of DXA images and our case definition; also the fact that the association between HBM and knee OA remained robust when restricted to those HBM cases with high hip BMD is reassuring, as hip OA is thought to have only a minimal influence on measured hip BMD [52]. Case–control studies are prone to selection bias; it is possible that less mobile individuals with OA were less likely to participate (or were selectively lost to follow-up in the ChS/HCS); however, such bias would be expected to affect both the HBM and control groups in the same direction. The lack of a standardised X-ray protocol across all centres may have reduced our sensitivity to detect differences in JSN between groups; this is likely to have particularly affected measured JSW in the HBM cases and family controls. [13]. Adjusting for BMI measured at a single time-point may have underestimated its effect on the HBM–OA association, as a previous study found that peak recalled body weight was superior to current BMI in predicting radiographic OA [53]. Finally, we cannot exclude residual confounding by factors such as physical activity which were not assessed in a consistent format across the different study populations.

In conclusion, our data support an association between HBM and an increased prevalence of radiographic knee OA predominantly characterised by osteophytosis. Taken together with our previous findings at the hip joint, this suggests that HBM individuals have a predisposition to a bone-forming phenotype of OA affecting multiple weight-bearing joint sites. In addition, BMI appears to be a partial mediator of the HBM–OA association at the knee, suggesting that HBM modifies the risk of knee OA via multiple pathways. Our findings add to existing evidence that increased BMD represents a risk factor for OA of the large joints, and suggest a mechanism involving an altered balance between bone formation and resorption.

## Funding

This work was supported by was supported by the Wellcome Trust and the NIHR CRN (portfolio number 5163) (study design and recruitment). CLG was funded through a Wellcome Trust Clinical Research Training Fellowship (080280/Z/06/Z). Ongoing support is being provided by Arthritis Research UK, who also fund SH through a Clinical PhD Studentship (grant ref 19580) and CLG through a Clinician Scientist Fellowship (grant ref 20000). The Hertfordshire cohort study is supported by the MRC, Arthritis Research UK and the NIHR Nutrition Biomedical Research Centre, University of Southampton. Funders had no role in the design, conduct or analysis of the present study.

## Competing interest statement

The authors declare no competing interests relevant to this work.

## Figures and Tables

**Table 1:** Semi-quantitative scoring of radiographic features of knee osteoarthritis. Grading of individual radiographic features (except chondrocalcinosis) was performed using an atlas [29]. KL (Kellgren–Lawrence) grades defined as 0 — no features of OA, 1 — doubtful osteophyte, 2 — definite osteophyte, 3 — definite osteophyte plus narrowing, 4 — osteophyte/narrowing/deformity as in Spector 1993 [34]. OA = osteoarthritis, OP = osteophyte, JSN = joint space narrowing.

OA feature	Categorical grading	Binary variable (s)
KL grade (global knee OA)	0–4	KL grade ≥ 2 (OA present), KL grade ≥ 3 (moderate OA)
Medial compartment osteophyte	0–3	Any osteophyte (any OP grade ≥ 1), moderate osteophyte (any OP grade ≥ 2)
Lateral compartment osteophyte	0–3	
Medial JSN	0–3	Any JSN (JSN grade ≥ 1), moderate JSN (JSN grade ≥ 2)
Lateral JSN	0–3	
Medial sclerosis	0–1	Subchondral sclerosis (grade ≥ 1)
Lateral sclerosis	0–1	
Medial chondrocalcinosis	0–1	Chondrocalcinosis (grade ≥ 1)
Lateral chondrocalcinosis	0–1	

**Table 2:** Demographics of study population. N for all variables is as shown except where indicated. HBM = high bone mass, ChS = Chingford 1000-women study, HCS = Hertfordshire cohort study, SD = standard deviation, BMI = body mass index, BMD = bone mineral density, L1–L4 = 1st to 4th lumbar vertebrae. BMD variables standardised according to scanner type (Hologic for Chingford/HCS controls, mixed Lunar/Hologic for HBM cases and family controls) using standard equations [54,55].

	HBM cases (N = 311)	Family controls (N = 183)	ChS controls (N = 599)	HCS controls (N = 209)	Combined controls (N = 991)
	Mean (SD)	Mean (SD)	Mean (SD)	Mean (SD)	Mean (SD)
Age (years)	60.8 (14.3)	54.0 (16.1)	62.1 (10.0)	75.2 (2.61)	63.4 (12.5)
BMI (kg/m^2^)	30.6 (5.83)	28.0 (4.77)	27.0 (4.79)	27.7 (4.34)	27.3 (4.71)
BMD total hip (g/cm^2^)	1.27^a^ (0.19)	1.00^c^ (0.14)	0.91^e^ (0.13)	0.97 (0.14)	0.94^g^ (0.14)
BMD L1–L4 (g/cm^2^)	1.55^b^ (0.19)	1.18^d^ (0.17)	1.04^f^ (0.17)	1.10 (0.20)	1.08^h^ (0.18)


	N (%)	N (%)	N (%)	N (%)	N (%)

Females	231 (74.3)	85 (46.5)	599 (100)	122 (58.4)	806 (81.3)

a N = 300.

b N = 299.

c N = 180.

d N = 181.

e N = 519.

f N = 583.

g N = 908.

h N = 973.

**Table 3:** Prevalence of knee OA features in HBM cases and control groups. P values refer to comparison with HBM cases. N for all variables is as shown except where indicated and refers to number of knee joints analysed. Quantitative measure of joint space width (JSW) limited to HBM study participants (HBM cases and family controls) only. p values from chi-squared test (binary outcomes)/unpaired t-test (continuous minimum JSW). ChS = Chingford 1000-women study, HCS = Hertfordshire cohort study, SD = standard deviation.

	HBM cases (N = 609)	Family controls (N = 362)		ChS controls (N = 1172)		HCS controls (N = 403)		Combined controls (N = 1937)	
	N (%)	N (%)	p	N (%)	p	N (%)	p	N (%)	p
Knee OA (KL ≥ 2)	192 (31.5)	46 (12.7)	< 0.001	234 (20.0)	< 0.001	124 (30.8)	0.799	404 (20.9)	< 0.001
Knee OA (KL ≥ 3)	81 (13.3)	22 (6.1)	< 0.001	87 (7.4)	< 0.001	64 (15.9)	0.251	173 (8.9)	0.002
Any osteophyte (≥ grade 1)	192 (31.5)	46 (12.7)	< 0.001	235 (20.1)	< 0.001	124 (30.8)	0.799	405 (20.9)	< 0.001
Osteophyte (≥ grade 2)	86 (14.1)	12 (3.3)	< 0.001	93 (7.9)	< 0.001	51 (12.7)	0.504	156 (8.1)	< 0.001
Any JSN (≥ grade 1)	91 (14.9)	37 (10.2)	0.035	149 (12.7)	0.191	95 (23.6)	0.001	281 (14.5)	0.791
JSN (≥ grade 2)	36 (5.9)	8 (2.2)	0.007	32 (2.7)	0.001	31 (7.7)	0.265	71 (3.7)	0.016
Subchondral sclerosis	18 (3.0)	6 (1.7)	0.208	28 (2.4)	0.475	8 (2.0)	0.339	42 (2.2)	0.264
Chondrocalcinosis	53 (8.7)	17 (4.7)	0.020	50 (4.3)	< 0.001	53 (13.2)	0.024	120 (6.2)	0.032


	Mean (SD)	Mean (SD)	p	Mean (SD)	p	Mean (SD)	p	Mean (SD)	p

Minimum JSW (medial), mm^a^	4.35 (1.18)	4.62 (1.09)	0.001	–	–	–	–	–	–

a N = 607 (HBM cases), 360 (family controls)

**Table 4:** GEE regression analysis of radiographic knee OA variables in HBM cases vs. all combined controls. Results show odds ratios (OR), with 95% confidence interval (95% CI). N (total no. of knee joints analysed) = 609 HBM cases, 1937 controls. Model 1 = unadjusted, model 2 = adjusted for age and gender. GEE = generalised estimating equations with logistic link function.

Outcome	Model	OR (95% CI) in HBM cases vs. controls	p value
Knee OA (KL ≥ 2)	1	1.76 (1.37, 2.27)	< 0.001
	2	2.38 (1.81, 3.14)	< 0.001
Knee OA (KL ≥ 3)	1	1.59 (1.14, 2.22)	0.006
	2	1.98 (1.39, 2.82)	< 0.001
Any osteophyte (≥ grade 1)	1	1.76 (1.37, 2.26)	< 0.001
	2	2.38 (1.80, 3.13)	< 0.001
Osteophyte (≥ grade 2)	1	1.89 (1.35, 2.64)	< 0.001
	2	2.40 (1.69, 3.41)	< 0.001
Any JSN (≥ grade 1)	1	1.05 (0.78, 1.43)	0.731
	2	1.18 (0.86, 1.62)	0.299
JSN (≥ grade 2)	1	1.71 (1.06, 2.75)	0.027
	2	1.95 (1.20, 3.18)	0.007
Subchondral sclerosis	1	1.42 (0.76, 2.63)	0.270
	2	1.66 (0.89, 3.11)	0.112
Chondrocalcinosis	1	1.42 (0.92, 2.20)	0.111
	2	1.65 (1.02, 2.66)	0.042

**Table 5:** Pattern of knee OA, summarised according to HBM case status, in combined population. Top panel shows compartment involvement in all knees with OA (defined as KL ≥ 2; at least one definite osteophyte) n = 169 HBM case knees, 390 control knees. Bottom panel shows compartment involvement in knees with KL ≥ 3 (osteophyte plus narrowing), n = 75 HBM case knees, 166 control knees. Knees of HBM cases/family controls with self-reported inflammatory arthritis (n = 12), and knees with appearances suggesting secondary OA (n = 2) excluded. Note that a small number of knees with KL grade ≥ 2 were missing the knee compartments variable (n = 23).

Compartment affected	Medial	Lateral	Both	p value^a^
	N (%)	N (%)	N (%)	
*Knees with KL grade ≥ 2*				
HBM cases	103 (61.0)	31 (18.3)	35 (20.7)	0.467
Controls	220 (56.4)	89 (22.8)	81 (20.8)	
All	323 (57.8)	120 (21.5)	116 (20.8)	

*Knees with KL grade ≥ 3 (i.e. knees with narrowing)*				
HBM cases	65 (86.7)	4 (5.3)	6 (8.0)	0.037
Controls	120 (72.3)	26 (15.7)	20 (12.1)	
All	185 (76.8)	30 (12.5)	26 (10.8)	

a p values from chi-squared test.
