# Supplementary material for: Individuals with high bone mass have an increased prevalence of radiographic knee osteoarthritis
Source: Bone. 2015 Feb;71:171–9. doi: 10.1016/j.bone.2014.10.015 (PMC4289915; doi:10.1016/j.bone.2014.10.015)
Supplement: Supplementary file 1 — Supplementary tables. [file mmc1.docx]

| **Outcome** | **Model** | **OR (95% CI) in HBM cases *vs.* controls** | **p value** |
| --- | --- | --- | --- |
| **Knee OA (KL≥2)** | 1 | 3.20 (2.07, 4.94) | <0.001 |
|  | 2 | 2.24 (1.40, 3.59) | 0.001 |
| **Knee OA (KL ≥3)** | 1 | 2.39 (1.35, 4.22) | 0.003 |
|  | 2 | 1.82 (0.99, 3.33) | 0.054 |
| **Any osteophyte (≥grade 1)** | 1 | 3.20 (2.07, 4.94) | <0.001 |
|  | 2 | 2.24 (1.40, 3.59) | 0.001 |
| **Osteophyte (≥grade 2)** | 1 | 4.81 (2.45, 9.43) | <0.001 |
|  | 2 | 3.79 (1.92, 7.48) | <0.001 |
| **Any JSN (≥grade 1)** | 1 | 1.58 (0.95, 2.61) | 0.078 |
|  | 2 | 1.16 (0.67, 2.01) | 0.590 |
| **JSN (≥grade 2)** | 1 | 2.83 (1.19, 6.75) | 0.019 |
|  | 2 | 2.46 (0.99, 6.10) | 0.053 |
| **Subchondral sclerosis** | 1 | 1.86 (0.64, 5.34) | 0.252 |
|  | 2 | 1.41 (0.50, 4.02) | 0.517 |
| **Chondrocalcinosis** | 1 | 1.89 (0.92, 3.91) | 0.085 |
|  | 2 | 1.32 (0.61, 2.86) | 0.488 |
| **Outcome** | **Model** | **β (95% CI)** | **p value** |
| **Minimum JSW (medial) (mm)^1^** | 1 | -0.28 (-0.48, -0.09) | 0.004 |
|  | 2 | 0.02 (-0.15, 0.20) | 0.817 |
| **Tibial plateau width (mm)** | 1 | -2.88 (-4.19, -1.57) | <0.001 |
|  | 2 | 0.23 (-0.65, 1.10) | 0.610 |

**Supplementary table 1: GEE regression analysis of radiographic knee OA variables in HBM cases *vs.* family / spouse controls.** Results show odds ratios (OR) or mean difference between groups, with 95% confidence interval (95% CI). N (total no. of knee joints analysed) = 609 HBM cases, 362 controls except where indicated. Model 1=unadjusted, model 2=adjusted for age and gender. GEE = generalised estimating equations with logistic / identity link function for binary / continuous outcomes. ^1^Medial compartment joint space width (JSW) N= 607 (HBM cases), 360 (family controls).

| **Outcome** | **Model** | **OR (95% CI) in HBM cases *vs.* controls** | **p value** |
| --- | --- | --- | --- |
| **Knee OA (KL≥2)** | 1 | 2.13 (1.58, 2.89) | <0.001 |
|  | 2 | 2.32 (1.68, 3.21) | <0.001 |
| **Knee OA (KL ≥3)** | 1 | 1.75 (1.13, 2.70) | 0.012 |
|  | 2 | 1.83 (1.17, 2.88) | 0.009 |
| **Any osteophyte (≥grade 1)** | 1 | 2.12 (1.57, 2.87) | <0.001 |
|  | 2 | 2.31 (1.67, 3.19) | <0.001 |
| **Osteophyte (≥grade 2)** | 1 | 1.86 (1.23, 2.82) | 0.003 |
|  | 2 | 1.98 (1.28, 3.06) | 0.002 |
| **Any JSN (≥grade 1)** | 1 | 1.09 (0.74, 1.61) | 0.668 |
|  | 2 | 1.09 (0.73, 1.62) | 0.665 |
| **JSN (≥grade 2)** | 1 | 1.78 (0.90, 3.53) | 0.099 |
|  | 2 | 1.82 (0.90, 3.66) | 0.094 |
| **Subchondral sclerosis** | 1 | 1.04 (0.45, 2.42) | 0.925 |
|  | 2 | 1.04 (0.44, 2.45) | 0.934 |
| **Chondrocalcinosis** | 1 | 1.91 (1.05, 3.45) | 0.033 |
|  | 2 | 2.10 (1.12, 3.96) | 0.021 |

**Supplementary table 2: GEE regression analysis of radiographic knee OA variables in HBM female cases *vs.* Chingford study female controls.** Results show odds ratios (OR), with 95% confidence interval (95% CI). N (total no. of knee joints analysed) =418 HBM cases, 1172 controls; HBM cases aged <40y (N=38 knees) were excluded. GEE = generalised estimating equations with logistic link function.

| **Outcome** | **Model** | **OR (95% CI) in HBM cases *vs.* controls** | **p value** |
| --- | --- | --- | --- |
| **Knee OA (KL≥2)** | 1 | 2.19 (1.45, 3.31) | <0.001 |
|  | 2 | 2.29 (1.49, 3.53) | <0.001 |
| **Knee OA (KL ≥3)** | 1 | 1.65 (1.03, 2.65) | 0.036 |
|  | 2 | 1.82 (1.13, 2.94) | 0.014 |
| **Any osteophyte (≥grade 1)** | 1 | 2.19 (1.45, 3.31) | <0.001 |
|  | 2 | 2.29 (1.49, 3.53) | <0.001 |
| **Osteophyte (≥grade 2)** | 1 | 2.34 (1.42, 3.87) | 0.001 |
|  | 2 | 2.44 (1.47, 4.05) | 0.001 |
| **Any JSN (≥grade 1)** | 1 | 1.07 (0.69, 1.66) | 0.773 |
|  | 2 | 1.19 (0.76, 1.86) | 0.457 |
| **JSN (≥grade 2)** | 1 | 1.52 (0.81, 2.85) | 0.194 |
|  | 2 | 1.62 (0.85, 3.06) | 0.141 |
| **Subchondral sclerosis** | 1 | 3.14 (1.17, 8.41) | 0.023 |
|  | 2 | 3.31 (1.24, 8.85) | 0.017 |
| **Chondrocalcinosis** | 1 | 1.39 (0.78, 2.45) | 0.260 |
|  | 2 | 1.52 (0.85, 2.72) | 0.156 |

**Supplementary table 3: GEE regression analysis of radiographic knee OA variables in HBM cases aged ≥65 years *vs.* HCS controls.** Results show odds ratios (OR), with 95% confidence interval (95% CI). N (total no. of knee joints analysed) = 239 HBM cases, 403 controls. GEE = generalised estimating equations with logistic link function.

| **Outcome** | **Model** | **OR (95% CI) in HBM cases *vs.* controls** | **p value** |
| --- | --- | --- | --- |
| **Knee OA (KL≥2)** | 1 | 1.66 (1.25, 2.20) | <0.001 |
|  | 2 | 2.27 (1.67, 3.10) | <0.001 |
| **Knee OA (KL ≥3)** | 1 | 1.27 (0.85, 1.91) | 0.241 |
|  | 2 | 1.70 (1.11, 2.60) | 0.015 |
| **Any osteophyte (≥grade 1)** | 1 | 1.65 (1.25, 2.19) | <0.001 |
|  | 2 | 2.26 (1.66, 3.08) | <0.001 |
| **Osteophyte (≥grade 2)** | 1 | 1.55 (1.05, 2.28) | 0.027 |
|  | 2 | 2.10 (1.39, 3.18) | <0.001 |
| **Any JSN (≥grade 1)** | 1 | 0.90 (0.62, 1.30) | 0.576 |
|  | 2 | 1.06 (0.73, 1.56) | 0.751 |
| **JSN (≥grade 2)** | 1 | 1.28 (0.68, 2.40) | 0.437 |
|  | 2 | 1.64 (0.86, 3.11) | 0.133 |
| **Subchondral sclerosis** | 1 | 0.99 (0.44, 2.24) | 0.986 |
|  | 2 | 1.32 (0.57, 3.03) | 0.515 |
| **Chondrocalcinosis** | 1 | 1.45 (0.84, 2.51) | 0.186 |
|  | 2 | 2.11 (1.17, 3.80) | 0.013 |

**Supplementary table 4: GEE regression analysis of radiographic knee OA variables in HBM female cases *vs.* all female controls.** Results show odds ratios (OR), with 95% confidence interval (95% CI). N (total no. of knee joints analysed) = 456 HBM cases, 1573 controls. Model 1=unadjusted, model 2=adjusted for age and gender. GEE = generalised estimating equations with logistic link function.

| **Outcome** | **Model** | **OR (95% CI) in HBM cases *vs.* controls** | **p value** |
| --- | --- | --- | --- |
| **Knee OA (KL≥2)** | 1 | 2.49 (1.41, 4.40) | 0.002 |
|  | 2 | 2.88 (1.55, 5.35) | 0.001 |
| **Knee OA (KL ≥3)** | 1 | 2.78 (1.45, 5.32) | 0.002 |
|  | 2 | 3.04 (1.53, 6.05) | 0.002 |
| **Any osteophyte (≥grade 1)** | 1 | 2.49 (1.41, 4.40) | 0.002 |
|  | 2 | 2.88 (1.55, 5.35) | 0.001 |
| **Osteophyte (≥grade 2)** | 1 | 3.82 (1.83, 7.96) | <0.001 |
|  | 2 | 4.05 (1.92, 8.53) | <0.001 |
| **Any JSN (≥grade 1)** | 1 | 1.45 (0.82, 2.55) | 0.198 |
|  | 2 | 1.52 (0.83, 2.76) | 0.174 |
| **JSN (≥grade 2)** | 1 | 2.65 (1.17, 6.00) | 0.019 |
|  | 2 | 2.60 (1.12, 6.02) | 0.026 |
| **Subchondral sclerosis** | 1 | 3.38 (0.97, 11.78) | 0.056 |
|  | 2 | 3.40 (0.97, 11.90) | 0.055 |
| **Chondrocalcinosis** | 1 | 1.16 (0.56, 2.38) | 0.688 |
|  | 2 | 1.11 (0.50, 2.47) | 0.794 |

**Supplementary table 5: GEE regression analysis of radiographic knee OA variables in HBM male cases *vs.* all male controls.** Results show odds ratios (OR), with 95% confidence interval (95% CI). N (total no. of knee joints analysed) =153 HBM cases, 364 controls. Model 1=unadjusted, model 2=adjusted for age and gender. GEE = generalised estimating equations with logistic link function.

| **Outcome** | **Model** | **OR (95% CI) in HBM cases *vs.* controls** | **p value** |
| --- | --- | --- | --- |
| **Knee OA (KL≥2)** | 1 | 1.76 (1.37, 2.27) | <0.001 |
|  | 2 | 2.38 (1.81, 3.14) | <0.001 |
|  | 3 | 1.62 (1.22, 2.16) | 0.001 |
| **Knee OA (KL ≥3)** | 1 | 1.59 (1.14, 2.22) | 0.006 |
|  | 2 | 1.98 (1.39, 2.82) | <0.001 |
|  | 3 | 1.29 (0.88, 1.89) | 0.189 |
| **Any osteophyte (≥grade 1)** | 1 | 1.76 (1.37, 2.26) | <0.001 |
|  | 2 | 2.38 (1.80, 3.13) | <0.001 |
|  | 3 | 1.62 (1.21, 2.15) | 0.001 |
| **Osteophyte (≥grade 2)** | 1 | 1.89 (1.35, 2.64) | <0.001 |
|  | 2 | 2.40 (1.69, 3.41) | <0.001 |
|  | 3 | 1.57 (1.09, 2.27) | 0.017 |
| **Any JSN (≥grade 1)** | 1 | 1.05 (0.78, 1.43) | 0.731 |
|  | 2 | 1.18 (0.86, 1.62) | 0.299 |
|  | 3 | 0.86 (0.62, 1.19) | 0.357 |
| **JSN (≥grade 2)** | 1 | 1.71 (1.06, 2.75) | 0.027 |
|  | 2 | 1.95 (1.20, 3.18) | 0.007 |
|  | 3 | 1.14 (0.67, 1.94) | 0.628 |
| **Subchondral sclerosis** | 1 | 1.42 (0.76, 2.63) | 0.270 |
|  | 2 | 1.66 (0.89, 3.11) | 0.112 |
|  | 3 | 1.20 (0.61, 2.35) | 0.594 |
| **Chondrocalcinosis** | 1 | 1.42 (0.92, 2.20) | 0.111 |
|  | 2 | 1.65 (1.02, 2.66) | 0.042 |
|  | 3 | 1.82 (1.12, 2.95) | 0.016 |

**Supplementary table 6:** **GEE regression analysis of radiographic knee OA variables in HBM cases *vs.* combined controls, with additional adjustment for BMI.** Results show odds ratios (OR) with 95% confidence interval (95% CI). N (total no. of knee joints analysed) = 609 HBM cases, 1937 controls. Model 1=unadjusted, model 2=adjusted for age and gender, model 3=adjusted for age, gender and BMI. GEE = generalised estimating equations with logistic link function.

| **Outcome** | **Exposure** | **Adjusted for** | **OR (95% CI) in HBM cases *vs.* controls** | **p value** |
| --- | --- | --- | --- | --- |
| Knee OA (KL ≥2) | HBM | Age, gender | 2.14 (1.20, 3.81) | 0.010 |
|  |  | Age, gender, BMI | 1.68 (0.91, 3.12) | 0.099 |
|  |  | Age, gender, height, weight | 1.70 (0.92, 3.15) | 0.093 |
|  |  | Age, gender, height, lean mass, fat mass | 1.71 (0.93, 3.15) | 0.086 |
|  |  | Age, gender, height, fat mass | 1.70 (0.92, 3.14) | 0.088 |
|  |  | Age, gender, height, lean mass | 1.95 (1.07, 3.54) | 0.028 |
|  |  | Age, gender, height, trunk fat | 1.80 (0.98, 3.28) | 0.057 |
|  |  | Age, gender, height, peripheral fat | 1.73 (0.94, 3.19) | 0.081 |
|  |  | Age, gender, height, android fat | 1.84 (1.02, 3.35) | 0.044 |
|  |  | Age, gender, height, gynoid fat | 1.83 (1.00, 3.35) | 0.051 |

**Supplementary table 7: Effect of adjusting for body composition variables on HBM-knee OA association in subpopulation of HBM case and family controls with total body DXA data.** N (no. of knees)= 373 (HBM cases), 239 (family controls). Analyses used generalised estimating equations with logistic link function to account for within-person clustering. Total body DXA data from Lunar and Hologic scanner types standardised using cross-calibration equations (see text).

| **Outcome** | **Model** | **OR (95% CI) in HBM cases *vs.* controls** | **p value** |
| --- | --- | --- | --- |
| **Knee OA (KL≥2)** | 1 | 1.76 (1.35, 2.30) | <0.001 |
|  | 2 | 2.50 (1.86, 3.37) | <0.001 |
| **Knee OA (KL≥3)** | 1 | 1.51 (1.08, 2.10) | 0.015 |
|  | 2 | 1.94 (1.35, 2.80) | <0.001 |
| **Any osteophyte (≥grade 1)** | 1 | 1.76 (1.35, 2.30) | <0.001 |
|  | 2 | 2.50 (1.86, 3.37) | <0.001 |
| **Osteophyte (≥grade 2)** | 1 | 1.92 (1.37, 2.68) | <0.001 |
|  | 2 | 2.54 (1.77, 3.66) | <0.001 |
| **Any JSN (≥grade 1)** | 1 | 1.06 (0.78, 1.44) | 0.709 |
|  | 2 | 1.21 (0.87, 1.67) | 0.256 |
| **JSN (≥ grade 2)** | 1 | 1.78 (1.12, 2.83) | 0.014 |
|  | 2 | 2.06 (1.26, 3.37) | 0.004 |
| **Subchondral sclerosis** | 1 | 1.44 (0.79, 2.63) | 0.237 |
|  | 2 | 1.70 (0.91, 3.20) | 0.097 |
| **Chondrocalcinosis** | 1 | 1.55 (1.01, 2.36) | 0.043 |
|  | 2 | 1.78 (1.12, 2.83) | 0.014 |

**Supplementary table 8: Person-level analysis of radiographic knee OA variables in HBM cases *vs.* combined controls.** Worst knee joint in each individual analysed. Results show odds ratios (OR), with 95% confidence interval (95% CI). N (total no. of knee joints analysed) =311 HBM cases, 991 controls. Model 1=unadjusted, model 2=adjusted for age and gender.

| **Outcome** | **Model** | **OR (95% CI) in HBM cases *vs.* controls** | **p value** |
| --- | --- | --- | --- |
| **KL ≥2** | 1 | 1.78 (1.39, 2.29) | <0.001 |
|  | 2 | 2.43 (1.85, 3.20) | <0.001 |
| **KL ≥3** | 1 | 1.64 (1.19, 2.26) | 0.003 |
|  | 2 | 2.07 (1.47, 2.93) | <0.001 |

**Supplementary table 9:** **GEE regression analysis of radiographic knee OA in HBM cases *vs.* combined controls, including knee replacements.** All total knee replacements (n=30) and unicompartmental knee replacements (n=2) classified as KL score 4. Model 1 = unadjusted, model 2 = adjusted for age and gender. GEE = generalised estimating equations with logistic link function. N (total no. of knee joints analysed) = 622 (HBM cases), 1956 (controls).
